# The Role of *De Novo* Variants in Patients with Congenital Diaphragmatic Hernia

**DOI:** 10.3390/genes12091405

**Published:** 2021-09-11

**Authors:** Charlotte Bendixen, Heiko Reutter

**Affiliations:** 1 Unit of Paediatric Surgery, Department of General, Visceral, Vascular and Thoracic Surgery, University Hospital Bonn, 53127 Bonn, Germany; 2 Institute of Human Genetics, University Hospital of Bonn, 53127 Bonn, Germany; reutter@uni-bonn.de; 3 Division of Neonatology and Paediatric Intensive Care, Department of Pediatrics and Adolescent Medicine, University Hospital Erlangen, 91054 Erlangen, Germany

**Keywords:** congenital diaphragmatic hernia, *de novo* variants, impaired reproductive fitness, mortality

## Abstract

The genetic etiology of congenital diaphragmatic hernia (CDH), a common and severe birth defect, is still incompletely understood. Chromosomal aneuploidies, copy number variations (CNVs), and variants in a large panel of CDH-associated genes, both *de novo* and inherited, have been described. Due to impaired reproductive fitness, especially of syndromic CDH patients, and still significant mortality rates, the contribution of *de novo* variants to the genetic background of CDH is assumed to be high. This assumption is supported by the relatively low recurrence rate among siblings. Advantages in high-throughput genome-wide genotyping and sequencing methods have recently facilitated the detection of *de novo* variants in CDH. This review gives an overview of the known *de novo* disease-causing variants in CDH patients.

## 1. Introduction

Congenital diaphragmatic hernia (CDH) is a relatively common birth defect reported to affect 2–3 per 10,000 live births [1]. Due to a high early neonatal and prenatal mortality, the hidden prevalence might be even higher [2]. The term CDH comprises a variety of defects in the diaphragm, ranging from diaphragmatic eventration to localized defects of variable size and locations to diaphragmatic agenesis. The most common type is the so-called “Bochdalek hernia” (dorsolateral) on the left side. CDH leads to herniation of abdominal viscera into the thorax during early embryonic development. Newborn patients typically present with respiratory distress which is, in short, due to hypoplasia of the lungs accompanied by abnormal structure of pulmonary vessels and alveolar septa, and pulmonary hypertension. Advancements in the prenatal diagnosis and postnatal management of CDH have led to reduced but still high mortality rates of 20–30% [3,4]. Surviving patients often exhibit significant long-term morbidity [5].

The etiology of CDH is incompletely understood. It is suggested that both genetic and environmental factors contribute to CDH, and although associations with different environmental factors have been described, no finding could be replicated to date [6]. From a medical genetics point of view, about 40% of CDH patients present syndromic. These patients present with additional anomalies of other organ systems (“non-isolated”), mostly cardiac defects, malformations of the central nervous system, urinary tract, and gastrointestinal system [7]. In these cases, a genetic diagnosis can be established more likely than in cases of isolated or non-syndromic CDH. Overall, in about 30% of CDH patients disease-causing genetic aberrations can be identified by chromosomal analysis, molecular karyotyping, and exome/or genome sequencing. Here, it has been shown that about 6% of CDH patients present with chromosomal imbalances detectable by routine chromosomal analysis or molecular karyotyping [8]. Earlier reports describe detection rates of up to 10% [9]. Using a customized array comparative genomic hybridization assay, Zhu et al. reported likely causative CNVs in 13% of a mixed CDH cohort [10]. An additional 3–10% of patients present with known monogenic syndromes. More recent sequencing studies have identified *de novo* damaging variants in known and novel CDH-associated genes in 10–30% of CDH patients [11,12,13,14,15,16]. Furthermore, is has been shown that the presence of a likely damaging *de novo* variant in a patient is associated with higher mortality and overall worse clinical outcome [17].

To establish a genetic diagnosis is increasingly important for affected families to provide proper counseling, especially as more CDH survivors reach reproductive age. This review focuses on the role of *de novo* events in CDH patients.

## 2. Known Genetic Factors

### 2.1. Associated Microscopic and Submicroscopic Anomalies

Except for the theoretical possibility of a trisomy 21 due to parental balanced translocation of chromosome 21 (not reported/investigated by most papers), all aneuploidies associated with CDH to date have been described to occur *de novo*. Aneuploidies (rarely) associated with CDH include trisomy 13, 18, 21, and triple X [17]. Furthermore it has been described in females with 45,X karyotype [18]. More frequently CDH has been described in patients with mosaic tetrasomy 12p (Pallister-Killian syndrome) [19], which always occurs *de novo*.

Other frequently detected CNVs include 15q26 deletion [20], comprising the CDH-associated gene *NR2F2* [21]; 8p23.1 deletion [22], comprising the CDH-associated gene *ZFPM2* [23]; 11q23 duplication typically resulting from parental balanced translocations [24], and 1q41–42 deletion [25], which includes the CDH-associated genes *HLX* and *DISP1* [26,27].

Less frequently described in association with CDH 4p16 deletions (Wolf-Hirschhorn syndrome) [28,29], comprising the CDH-associated gene *FGFRL1* [30]; 22q11.2 deletion [31]; deletion and duplication of 17q12 [32,33], and 1q12 duplication [34]. Very rare CNVs in CDH patients have been described and comprehensively been reviewed by Wynn et al. [18].

Among the CNVs found in CDH patients are, as expected, many *de novo* events. Other CNVs are caused by unbalanced translocations from a parental balanced translocation. Few CNVs are reported to be inherited [32,35]. The genome-wide *de novo* CNV rate in general is estimated to be 0.5–3% [36,37], about 2–12 times lower than the rate of *de novo* CNVs in CDH patients. CNVs are more likely to be detected in non-isolated cases of CDH than in isolated cases [8] and in general, more deletions (with a pathomechanism of haploinsufficency for CDH-associated genes) have been reported. Overall, *de novo* CNVs have been shown to be a major contributor to the formation of CDH.

### 2.2. De Novo Variants in Monogenic CDH Syndromes

More than 20 syndromes with known genetic causes have been associated with the occurrence of CDH. Among these are dominant, recessive, and X-linked inherited syndromes. *de novo* events commonly play a role in autosomal dominant or X-linked syndromes. The rare occurrence of *de novo* events leading to a recessive CDH-associated syndrome is described for Cutis laxa Type 1C [38]. Some well-known monogenic syndromes caused by *de novo* events and featuring CDH are Cornelia de Lange syndrome (*NIPBL*) [39,40]; Craniofrontonasal syndrome (*EFNB1*) [41]; Focal dermal hypoplasia (*PORCN*) [42]; and Kabuki syndrome (*KMT2D*; *MLL2*) [14,43,44]. A full list of monogenic syndromes in which *de novo* events are reported is provided in Table 1. It has to be noted that for many described variants in other CDH-related autosomal dominant inherited syndromes, the inheritance pattern is not investigated or reported, but appears to be likely dominant *de novo*.

### 2.3. De Novo Variants in Non-Isolated CDH

Several genes harboring *de novo* variants in non-isolated CDH patients have been identified, most of them by whole exome (WES)/whole genome (WGS) sequencing techniques. Among these are some well-known CDH-associated genes. *de novo* variants in *GATA4* have been described in non-isolated [17,22,56] and isolated CDH [57]. *GATA4* is known to be associated with congenital heart defects in humans and is further supported by a mouse model [58]. It encodes a transcription factor that is part of the retinoic acid signaling pathway, which has been implicated in diaphragm development [59].

Repeatedly, non-isolated CDH patients were found to carry *de novo* variants in *NR2F2* [16,17,21,57], an interaction partner of *ZFPM2*, a gene commonly affected by the deletion of 8p23.1 observed in CDH patients. The role of *NR2F2* in diaphragm development is further supported by its expression pattern and a mouse model [60]. More recently, *de novo* variants in *MYRF*, a membrane associated transcription factor, have been described in non-isolated CDH patients, also showing cardiac and genitourinary malformations [12,17,61,62,63].

Other genes with described *de novo* variants in non-isolated CDH patients are listed in Table 2. Clinical features of patients are available in Table S1. In very few genes, variants in more than one patient could be detected. This illustrates the heterogeneity of the genetic background of CDH. The largest WES/WGS study on family trios could identify *de novo* likely gene-disrupting (LGD) or deleterious missense (D-mis) variants in 21% of non-isolated CDH cases [12]. Another family trio study also showed an increased burden of *de novo* D-mis and LGD variants in a mixed cohort of isolated and non-isolated CDH [13]. Recently a WES study established a genetic diagnosis in 28/76 (37%) non-isolated CDH patients, of which 15/76 (20%) were attributable to *de novo* variants [14]. These findings further strongly support a major role of *de novo* variants in CDH.

### 2.4. De Novo Variants in Isolated CDH

In patients with isolated CDH a genetic cause is less likely to be established by current genotyping or sequencing techniques. The above-mentioned study on case-parent-trios could identify *de novo* likely gene-disrupting or deleterious missense variants in only 12% of isolated CDH cases [12]. Among the described *de novo* variants in isolated CDH are variants in the already mentioned genes *ZFPM2* [12,23,68], *GATA4* [57], and *PTPN11* [12,16,17]. As in non-isolated CDH, variants in very few genes could be implicated in more than one patient. A list of genes with *de novo* variants in isolated CDH is provided in Table 3. Notably, some genes are reported to carry *de novo* variants in non-isolated and isolated CDH patients.

## 3. Discussion

Based on the current knowledge, we have to assume that *de novo* events play a major role in CDH etiology. In up to 30% of CDH cases a genetic cause can be established, more often in non-isolated than in isolated CDH. For the estimation of the fraction of causal CNVs/variants being *de novo*, large family trio studies are needed. However, in these, often only *de novo* events are reported. By looking at subsets of two large CNV studies [8,10] the fraction of causal CNVs being *de novo* can be estimated up to 80%. Similarly, the fraction of causal variants being *de novo* could be estimated around 50% [15]. However, these estimations are based on small sample sizes only. Most likely, the fraction of *de novo* events is currently underestimated due to restricted genetic testing for newborns with (especially sporadic isolated) CDH in clinical practice.

The contribution of *de novo* variants to a disease depends on several factors. (i) It is higher in sporadic than in familial diseases; (ii) it is higher when the impact on fitness of the disease is higher; (iii) it is higher in monogenic than in complex diseases [69]. On the other hand, the incidence of a disease caused by *de novo* events increases with (i) mutational target size; (ii) target mutability and (iii) paternal age at conception [69]. When conferring this to CDH, CDH is a mostly sporadic disease with high impact on fitness with not fully understood genetics, but monogenic forms being reported. The mutational target size is most likely large due to the heterogeneity of CDH. Paternal age at conception has not been reported to be a risk factor for CDH.

A well-studied example of a condition with reduced reproductive fitness is developmental delay/intellectual disability (DD/ID). Here it could be shown that *de novo* variants account for ~50% of the genetic background of DD/ID [70]. For CDH, a similar or even higher proportion can be hypothesized. Larger whole genome/whole exome sequencing studies on case-parent-trios will most likely reveal additional *de novo* variants. The pathogenicity of the many rare *de novo* variants reported in CDH patients could also be further supported by larger resequencing studies which would identify additional patients harboring the same variant.

Genetic counseling for affected families with the sporadic occurrence of non-syndromic CDH should however, imply the recurrence risk of about 1% in future pregnancies. This, however, changes accordingly, when a genetic diagnosis has been established. Regardless of the establishment of a genetic diagnosis, affected families should be referred to a prenatal medicine center during the first and second trimester of subsequent pregnancies.

## 4. Conclusions

Among rare and severe birth defects, CDH is one of the more common ones. The current knowledge on the genetics of CDH suggests that a substantial fraction of CDH is due to underlying genetic *de novo* events. However, it is conceivable that several common variants form a “risk haplotype” that predisposes to non-syndromic CDH.

## Figures and Tables

**Table 1:** Monogenic syndromes with associated CDH caused by *de novo* events.

Syndrome	OMIM	Gene	Chromosomal Location	Genomic Coordinates (GRCh38/hg38)	Additional Malformations	References
PDAC syndrome	#615524	*RARB*	3p24.3	chr3: 25,428,263–25,597,932	Micro-/Anophtalmia, pulmonary hypoplasia, cardiac abnormalities	[45]
Cornelia de Lange syndrome	#122470	*NIPBL*	5p13.2	chr5: 36,876,769–37,066,413	Hypertelorism, synophrys, low anterior hairline, upper limb malformations	[40,46,47]
Coffin-Siris syndrome	#135900, #614609	*ARID1B, SMARCA4*	6q25.3	chr6: 156,776,020–157,210,779 chr19: 10,961,001–11,062,256	Growth retardation, long eyelashes, frequent respiratory tract infections, hypotonia, developmental delay	[14,48]
Congenital heart defects and skeletal malformations syndrome (CHDSKM)	#617602	*ABL1*	9q34.12	chr9: 130,713,016–130,885,683	Dysmorphic facial features, congenital heart disease, skeletal abnormalities, joint laxity, failure to thrive, gastrointestinal problems, male genital anomalies	[14,49]
Apert syndrome	#101200	*FGFR2*	10q26.13	chr10: 121,479,857–121,598,403	Acrocephaly, micrognathia, limb malformations	[50]
Denys-Drash syndrome, Meacham syndrome	#194080, #608978	*WT1*	11p13	chr11: 32,389,058–32,435,360	Male pseudohermaphroditism, cardiac abnormalities	[51,52]
Kabuki syndrome	#147920	*KMT2D*	12q13.12	chr12: 49,018,978–49,060,794	Mental retardation, short stature, eversion of eyelids, finger pads	[14,43,44,53]
Marfan syndrome Type 1	#154700	*FBN1*	15q21.1	chr15: 48,408,313–48,645,709	Congenital contractures, arachnodactyly, aortic dilatation, cardiac valve insufficiency	[14,54]
Geleophysic dysplasia 2	#614185	*FBN1*	15q21.1	chr15: 48,408,313–48,645,709	Short stature, cardiac valvular thickening, skin thickening, joint problems	[17]
Rubinstein-Taybi syndrome 2	#613684	*EP300*	22q13.2	chr22: 41,092,592–41,180,077	Failure to thrive, cardiovascular abnormalities, motor and speech delays, dysmorphic facial features	[14,55]
Focal dermal hypoplasia	#305600	*PORCN*	Xp11.23	chrX: 48,508,992–48,520,808	Sparse hair, anophtalmia, limb malformations, Pentalogy of Cantrell	[42]
Craniofrontonasal syndrome	#304110	*EFNB1*	Xq13.1	chrX: 68,829,021–68,842,160	Coronal craniosynostosis, duplex thumb, partial agenesis of corpus callosum	[41]

**Table 2:** Genes with *de novo* variants in non-isolated CDH patients.

Gene	Chromosomal Location	Genomic Coordinates (GRCh38/hg38)	Number of Patients with *de novo* Variants	References	Design/Method of Studies
*PRKACB*	1p31.1	chr1: 84,078,062–84,238,498	1	[14]	trio WES
*SLC5A9*	1p33	chr1: 48,222,716–48,248,638	1	[14]	trio WES
*ZNF362*	1p35.1	chr1: 33,256,492–33,300,719	1	[17]	trio WES/WGS
*HSPG2*	1p36.12	chr1: 21,822,244–21,937,310	1 °	[17]	trio WES
*UBAP2L*	1q21.3	chr1: 154,220,955–154,270,847	1	[17]	trio WGS
*POGZ*	1q21.3	chr1: 151,402,724–151,459,494	1	[12]	clinical WES
*DISP1*	1q41	chr1: 222,815,039–223,005,995	1	[27]	targeted sanger sequencing
*INHBB*	2q14.2	chr2: 120,346,136–120,351,803	1	[14]	trio WES
*TTC21B*	2q24.3	chr2: 165,873,362–165,953,776	1	[17]	trio WGS
*ROBO1*	3p12.3	chr3: 78,598,688–79,019,015	1	[17]	targeted panel sequencing
*FOXP1*	3p13	chr3: 70,954,708–71,583,978	1	[15]	clinical WES
*RAF1*	3p25.2	chr3: 12,583,601–12,664,117	1	[12]	trio WES/WGS
*FAT4*	4q28.1	chr4: 125,314,955–125,492,932	1	[17]	trio WGS
*CDO1*	5q22.3	chr5: 115,804,733–115,816,659	1	[14]	trio WES
*FOXP4*	6p21.1	chr6: 41,546,426–41,602,384	1	[12]	trio WES/WGS
*PTPN12*	7q11.23	chr7: 77,537,295–77,640,069	1	[14]	trio WES
*BRAF*	7q34	chr7: 140,719,327–140,924,810	1	[12]	trio WES/WGS
*GATA4*	8p23.1	chr8: 11,704,202–11,760,002	3	[17,22,56]	targeted sanger sequencing, trio WGS
*EYA1*	8q13.3	chr8: 71,197,511–71,548,061	1	[11,57]	WES, targeted panel sequencing
*TLN1*	9p13.3	chr9: 35,696,948–35,732,195	1 °	[17]	trio WES
*PLPP6*	9p24.1	chr9: 4,662,294–4,665,258	1	[14]	trio WES
*NOTCH1*	9q34.3	chr9: 136,494,433–136,546,048	1	[17]	trio WGS
*CTR9*	11p15.3	chr11: 10,751,246–10,779,746	1 *	[16]	trio WES
*MYRF*	11q12.2	chr11: 61,752,636–61,788,518	11	[12,17,61,62,63]	trio WES/WGS, clinical WES, trio WGS
*PTPN11*	12q24.13	chr12: 112,419,112–112,504,764	1	[12]	trio WES/WGS
*HNRNPC*	14q11.2	chr14: 21,210,613–21,269,421	1	[17]	trio WGS
*BMP4*	14q22.2	chr14: 53,949,736–53,956,825	1	[64]	targeted sanger sequencing
*DLST*	14q24.3	chr14: 74,881,916–74,903,743	1	[14]	trio WES
*TCF12*	15q21.3	chr15: 56,918,644–57,289,853	1	[15]	clinical WES
*SIN3A*	15q24.2	chr15: 75,370,933–75,455,783	1	[14]	trio WES
*NR2F2*	15q26.2	chr15: 96,330,700–96,340,258	4	[16,17,21,57,65]	clinical WES, targeted panel sequencing, trio WES, trio WGS
*TRAF7*	16p13.3	chr16: 2,155,782–2,178,129	1	[15]	clinical WES
*ANKRD11*	16q24.3	chr16: 89,285,175–89,490,318	1	[17]	trio WGS
*MYH10*	17p13.1	chr17: 8,474,207–8,630,761	1	[66]	clinical WES
*TP53*	17p13.1	chr17: 7,668,421–7,687,490	1 *	[16]	trio WES
*NLK*	17q11.2	chr17: 28,042,677–28,196,381	1	[17]	trio WGS
*FZD2*	17q21.31	chr17: 44,557,484–44,561,262	1	[32]	aCGH
*ATXN7L3*	17q21.31	chr17: 44,191,805–44,198,070	1	[17]	trio WGS
*ALYREF*	17q25.3	chr17: 81,887,835–81,891,586	1	[12]	trio WES/WGS
*GATA6*	18q11.2	chr18: 22,169,589–22,202,528	1	[67]	trio WES
*NACC1*	19p13.13	chr19: 13,118,264–13,141,147	1	[12]	trio WES/WGS
*LONP1*	19p13.3	chr19: 5,691,835–5,720,572	1	[14]	trio WES
*LTBP4*	19q13.2	chr19: 40,601,369–40,629,818	1	[38]	targeted sanger sequencing
*ZC3H4*	19q13.32	chr19: 47,064,187–47,113,776	1	[12]	trio WES/WGS
*PCNA*	20p12.3	chr20: 5,114,953–5,126,626	1	[12]	trio WES/WGS
*EPB41L1*	20q11.23	chr20: 36,092,712–36,230,343	1	[12]	trio WES/WGS
*ARFGEF2*	20q13.13	chr20: 48,921,711–49,036,693	1	[14]	trio WES
*ADNP*	20q13.13	chr20: 50,888,918–50,931,437	1	[17]	trio WGS
*SCAF4*	21q22.11	chr21: 31,671,000–31,732,118	1	[17]	trio WGS
*DDX3X*	Xp11.4	chrX: 41,333,348–41,350,287	1	[15]	clinical WES
*USP9X*	Xp11.4	chrX: 41,085,445–41,236,579	1 °	[17]	trio WES/WGS
*CLCN4*	Xp22.2	chrX: 10,156,975–10,237,660	1	[14]	trio WES
*HCCS*	Xp22.2	chrX: 11,111,301–11,123,078	1	[15]	clinical WES
*STAG2*	Xq25	chrX: 123,961,314–124,102,656	1	[14]	trio WES

* Variants reported in the same patient, additionally *de novo* CNV deletion 8p23. ° Variants reported in the same patient.

**Table 3:** Genes with *de novo* variants in isolated CDH patients.

Gene	Chromosomal Location	Genomic Coordinates (GRCh38/hg38)	Number of Patients with *de novo* Variants	References	Design/Method of Studies
*HSPG2*	1p36.12	chr1: 21,822,244–21,937,310	2	[13,14]	trio WES
*ATAD3A*	1p36.33	chr1: 1,512,175–1,534,685	1	[12]	trio WES/WGS
*POGZ*	1q21.3	chr1: 151,402,724–151,459,494	1	[12]	trio WES/WGS
*KDM5B*	1q32.1	chr1: 202,724,495–202,808,421	1	[12]	trio WES/WGS
*ZBTB18*	1q44	chr1: 244,051,283–244,057,476	1	[12]	trio WES/WGS
*MYT1L*	2p25.3	chr2: 1,789,124–2,331,348	1	[12]	trio WES/WGS
*FOXP1*	3p13	chr3: 70,954,708–71,583,978	1	[12]	trio WES/WGS
*SRGAP3*	3p25.3	chr3: 8,980,594–9,249,213	1	[12]	trio WES/WGS
*KPNA1*	3q21.1	chr3: 122,421,902–122,514,939	1	[17]	trio WGS
*NAA15*	4q31.1	chr4: 139,301,505–139,391,384	1	[12]	trio WES/WGS
*SMO*	7q32.1	chr7: 129,188,633–129,213,545	1	[12]	trio WES/WGS
*GATA4*	8p23.1	chr8: 11,704,202–11,760,002	1	[57]	targeted panel sequencing
*ZFPM2*	8q23.1	chr8: 105,318,438–105,804,539	3	[12,23,68]	WES, trio WES/WGS, targeted sanger sequencing
*EMX2*	10q26.11	chr10: 117,542,746–117,549,546	1	[12]	trio WES/WGS
*WT1*	11p13	chr11: 32,389,058–32,435,360	3	[12,16]	trio WES/WGS
*PTPN11*	12q24.13	chr12: 112,419,112–112,504,764	3	[12,16,17]	trio WES/WGS
*MEIS2*	15q14	chr15: 36,889,204–37,100,549	1	[12]	trio WES/WGS
*TBX6*	16p11.2	chr16: 30,085,793–30,091,924	1	[11]	WES
*CTCF*	16q22.1	chr16: 67,562,467–67,639,176	1	[17]	trio WGS
*AP1G1*	16q22.2	chr16: 71,729,000–71,808,834	1	[12]	trio WES/WGS
*MYH10*	17p13.1	chr17: 8,474,207–8,630,761	1	[17]	targeted panel sequencxing
*SRSF1*	17q22	chr17: 58,000,919–58,007,246	1	[17]	trio WGS
*LONP1*	19p13.3	chr19: 5,691,835–5,720,572	2	[17]	trio WGS
*CIC*	19q13.2	chr19: 42,268,537–42,295,796	1	[12]	trio WES/WGS
*LAMA5*	20q13.33	chr20: 62,309,065–62,367,312	1	[12]	trio WES/WGS
*DIDO1*	20q13.33	chr20: 62,877,738–62,937,952	1	[12]	trio WES/WGS
*HSD17B10*	Xp11.22	chrX: 53,431,261–53,434,370	1	[12]	trio WES/WGS
*FLNA*	Xq28	chrX: 154,348,529–154,371,283	1	[17]	trio WGS

## Data Availability

Not applicable.

## Supplementary Materials

The following are available online at https://www.mdpi.com/article/10.3390/genes12091405/s1, Table S1: Additional clinical features of non-isolated CDH patients from Table 2.
