# Supplementary material for: The Role of De Novo Variants in Patients with Congenital Diaphragmatic Hernia
Source: Genes (Basel). 2021 Sep 11;12(9):1405. doi: 10.3390/genes12091405 (PMC8466043; doi:10.3390/genes12091405)
Supplement: Supplementary file 1 [file genes-12-01405-s001.zip › genes-1343491-supplementary.pdf]

Table S1. Additional clinical features of non-isolated CDH patients from Table 2.

Gene	Number of patients with *de novo* variants	Additional malformations	References
PRKACB	1	ventricular septum defect (VSD)	[14]
SLC5A9	1	Tetralogy of Fallot (ToF), intestinal malrotation, single umbilical artery	[14]
ZNF362	1	NR	[17]
HSPG2	1°	Partial agenesis of the corpus callosum, motor, language and cognitive delay	[17]
UBAP2L	1	NR	[17]
POGZ	1	microcephaly, seizures, dysmorphic ears, micropenis, facial dysmorphism, optic nerve hypoplasia	[12]
DISP1	1	ventricular septal defect and abnormal aorta, left-sided cleft lip with bilateral cleft palate, hypotonia, short neck, torticollis, scoliosis, bilateral single transverse palmar creases, wide thumbs	[27]
INHBB	1	congenital hydrocephalus, motor delay	[14]
TTC21B	1	NR	[17]
ROBO1	1	NR	[17]
FOXP1	1	horseshoe kidney, mildly hypoplastic left sided heart structures.	[15]
RAF1	1	bronchopulmonary sequestration	[12]
FAT4	1	NR	[17]
CDO1	1	anomalous hepatic veins, partial anomalous pulmonary venous return	[14]
FOXP4	1	C1 fused to skull, plagiocephaly	[12]
PTPN12	1	ear abnormality, micrognathia	[14]
BRAF	1	single umbilical artery	[12]
GATA4	3	[17] NR; [22] VSD; [56] atrial septal defect and ventricular septal defect, mild motor and cognitive delay	[17], [22], [56]
EYA1	1	[11] microtia and hearing loss; [57] NR	[11], [57]
TLN1	1°	partial agenesis of the corpus callosum, global developmental delay	[17]
PLPP6	1	scapula deformity, syndactyly, severe developmental delay	[14]
NOTCH1	1	NR	[17]
CTR9	1*	atrioventricular canal defect	[16]

MYRF	11	[12] atrial septum defect (ASD), VSD , ToF, bilateral undescended testes; VSD, no internal genital organs, blind-ending vagina, accessory spleen; ASD, VSD; hypoplastic left heart, ambiguous genitalia, undescended testes, intellectual disability and motor delay; Scimitar syndrome; [17] NR; [61] Scimitar syndrome, hypoplastic left heart syndrome, mitral valve atresia, hypoplastic aortic valve, VSD, ASD, PDA; Hypoplastic left heart syndrome, hypospadias, cryptorchidism; ambiguous genitalia, hypospadias, horseshoe kidney; [62] Scimitar syndrome, persistent urachus, undescended testis, cleft spleen, thymic involution, thyroid fibrosis; [63] Scimitar syndrome, aortic arch hypoplasia, ASD, bicuspid aortic valve, hypoplastic left heart syndrome, mitral stenosis, VSD, undescended testis, lung hypoplasia	[12], [17], [61], [62], [63]
PTPN11	1	univentricular defect	[12]
HNRNPC	1	NR	[17]
BMP4	1	anophthalmia, microphthalmia with sclerocornea, hydrocephalus	[64]
DLST	1	micrognathia, cleft palate, hemivertebrae	[14]
TCF12	1	asymmetric ventriculomegaly, hypotonia, hyporeflexia, club feet, cryptorchidism, mild dysmorphic features, ulnar deviation of wrist, talipes equinovarus.	[15]
SIN3A	1	pelvic kidney, palate defect, cognitive deficits	[14]
NR2F2	4	[17] ASD; [57] NR; [16] aortic arch hypoplasia, intrauterine growth restriction; [21] ASD, persistent foramen ovale, mild developmental delay; [65] hypoplastic left heart; severely dilated right ventricle, dysorder of sex development with virilisation, gonads not palpable	[16], [17], [21], [57], [65]
TRAF7	1	intellectual disability, structural brain abnormalities, hypotonia, hearing loss, optic atrophy, aortic regurgitation, pain insensitivity, bilateral chronic lower leg lymphedema, small penis, undecended testicle, hepatomegaly, enlarged bladder with cyst, hypothyroidism, biateral flat feet, short stature, blepharophimosis	[15]
ANKRD11	1	NR	[17]
MYH10	1	intrauterine growth restriction, microcephaly, developmental delay, failure to thrive, congenital bilateral hip dysplasia, cerebral and cerebellar atrophy, hydrocephalus	[66]
TP53	1*	atrioventricular canal defect	[16]
NLK	1	NR	[17]
FZD2	1		[32]
ATXN7L3	1	NR	[17]
ALYREF	1	VSD	[12]
GATA6	1	ToF, single umbilical artery	[67]
NACC1	1	pyloric stenosis	[12]
LONP1	1	VSD	[14]
LTBP4	1	hypotonia, poor feeding, skin laxity, retrognathia, umbilical hernia, widely spaced first and second toes, unilateral transverse palmar crease, pyloric stenosis, tracheomalacia	[38]

ZC3H4	1	thinning of corpus callosum	[12]
PCNA	1	neurodevelopmental disorder, left middle cerebral artery stroke	[12]
EPB41L1	1	microcephaly, siezures, dysmorphic ears, micropenis, facial dysmorphism, optic nerve hypoplasia	[12]
ARFGEF2	1	Overlapping toes, camptodactly, global developmental delay	[14]
ADNP	1	NR	[17]
SCAF4	1	NR	[17]
DDX3X	1	cystic hygroma, cutaneous edema	[15]
USP9X	1°	partial agenesis of the corpus callosum, global developmental delay	[17]
CLCN4	1	bilateral hip dysplasia, failure to thrive, motor and speech delay	[14]
HCCS	1	agenesis of corpus callosum, bilateral adducted thumbs	[15]
STAG2	1	microcephaly, sacral dimple, scoliosis, intellecutal impairment	[14]

NR: not reported
